# Challenges during the execution, results, and monitoring phases of ecological restoration: Learning from a country-wide assessment

**DOI:** 10.1371/journal.pone.0249573

**Published:** 2021-04-06

**Authors:** Moisés Méndez-Toribio, Cristina Martínez-Garza, Eliane Ceccon

**Affiliations:** 1 Red de Diversidad Biológica del Occidente Mexicano, Centro Regional del Bajío, Instituto de Ecología, Michoacán, México; 2 Centro de Investigación en Biodiversidad y Conservación (CIByC), Universidad Autónoma del Estado de Morelos (UAEM), Cuernavaca, México; 3 Centro Regional de Investigaciones Multidisciplinarias, Universidad Nacional Autónoma de México, Cuernavaca, México; Feroze Gandhi Degree College, INDIA

## Abstract

Outcomes from restoration projects are often difficult for policymakers and stakeholders to assess, but this information is fundamental for scaling up ecological restoration actions. We evaluated technical aspects of the interventions, results (ecological and socio-economic) and monitoring practices in 75 restoration projects in Mexico using a digital survey composed of 137 questions. We found that restoration projects in terrestrial ecosystems generally relied on actions included in minimal (97%) and maximal (86%) intervention, while in wetlands, the preferred restoration strategies were intermediate (75%) and minimal intervention (63%). Only a third of the projects (38%) relied on collective learning as a source of knowledge to generate techniques (traditional management). In most of the projects (73%), multiple criteria (>2) were considered when selecting plant species for plantings; the most frequently used criterion was that plant species were found within the restoration area, native or naturalized (i.e., a *circa situm* criterion; 88%). In 48% of the projects, the biological material required for restoration (e.g., seeds and seedlings) were gathered or propagated by project implementers rather than purchased commercially. Only a few projects (between 33 and 34%) reached a high level of biodiversity recovery (>75%). Most of the projects (between 69 to71%) recovered less than 50% of the ecological services. Most of the projects (82%) led to improved individual relationships. The analysis revealed a need to implement strategies that are cost-effective, the application of traditional ecological knowledge and the inclusion of indigenous people and local communities in restoration programs at all stages—from planning to implementation, through monitoring. We also identified the need to expand research to develop effective tools to assess ecosystems’ regeneration potential and develop theoretical frameworks to move beyond short-term markers to set and achieve medium- and long-term goals. Cautious and comprehensive planning of national strategies must consider the abovementioned identified gaps.

## Introduction

Restoration intervention is implemented to restore damaged ecosystems. Minimal intervention, called also “passive restoration” [1] or “unassisted forest regeneration” [2], includes actions to remove chronic disturbance for allowing natural succession to proceed [3]. This intervention is considered cost-effective for large-scale restoration [4]. Fostering natural succession requires low levels of disturbance at the landscape or local level [5]. Intermediate restoration or assisted natural regeneration (ANR) aims to accelerate the natural succession process by removing sources of disturbance (e.g., fire, grazing, or wood harvesting) or reducing impediments to natural forest succession [e.g., soil degradation, competition with weedy species, lack of seeds; 6,7]. The ANR technique is usually a simple, less expensive than maximal intervention, and effective for converting areas of degraded vegetation to more productive forests [8]. The implementation of ANR is suggested at intermediate levels of degradation [7]. Finally, maximal intervention involves establishing restoration plantings, including enrichment planting [3]: establishment of valuable timber species in species poor forest [9]. This approach is the most effective for recovering biodiversity, but it is usually an expensive technique [10,11]. Defining which level of intervention to apply is a difficult task [12]. For instance, before plantings are established, it is frequently necessary to construct civil infrastructure for erosion control [13] or assess chronic disturbance by establishing an initial diagnosis of the degraded ecosystem [7,14]. In addition, although Traditional Ecological Knowledge (TEK) from local communities, such as Indigenous Peoples and Local Communities (IPLCs), has been recognized to have a valuable contribution to projects [15,16], TEK is currently almost never considered in restoration programs [17–21]. Restoration plantings involve the challenge of finding seeds with high genetic diversity [22] and selecting species with high performance in the context of climate change [23,24]. Criteria for selecting planting material, however, are often poorly related to restoration objectives [21] or even overlooked by practitioners [25]. Tree species for assisted succession are selected for their availability in nurseries [26] or some desired traits (e.g., abundant foliage, good root development, resistance to pests) rather than for their role in a particular ecological process or ecosystem function [25]. Projects that establish plantings to increase primary productivity, pollination services, or seed dispersal are indeed scarce [27–29]. In summary, the adequate selection of the level of restoration intervention depends on previous ecological knowledge, degree of disturbance, objectives, available budget, and restoration scale.

After planning and executing interventions, results, progress, and effectiveness should be evaluated [30]. Monitoring enables assessing restoration outcomes compared to initial ecosystem conditions or to a reference ecosystem [14]. Evaluate restoration progress allows knowing whether objectives have been achieved [31]. In addition, collaborative and cross-scalar monitoring generates valuable information for social learning and adaptive management [32], which is an iterative process allowing projects to adapt to unexpected threats and to learn from the process [30,33] There is still, however, a strong debate about what variables should be monitored [34–37] given that some projects can take decades before showing results [38,39]. In practice, monitoring should include biophysical and socio-economic variables [40] and a multi-scalar, multi-site monitoring approach is needed [31]. Nevertheless, projects that have a holistic vision are scarce, and the contribution that IPLCs can make to assess the progress of projects is still insufficiently studied [16]. Evidence to date about monitoring of Latin American restoration projects indicates a lack of forecast and proper planning based on a clear conceptual framework [33]. For example, in Colombia, 50% of projects did not include medium- or long-term variables, and only 5% included social variables [41]. Adequate monitoring is fundamental for determining whether restoration projects are reaching their goals and for verifying their overall effectiveness and success.

Because of the magnitude of the degradation and destruction of ecosystems in Latin America and worldwide [42], the restoration of ecosystems and landscapes is an international priority. The Aichi Target 15 of the Convention on Biological Diversity called countries to restore 15% of degraded or deforested forests, highlighting the recovery of biodiversity [43]. The Bonn Challenge, launched in 2011 by Germany and the IUCN, is a global effort to restore 150 million hectares of the world’s deforested and degraded land by 2020, as well as 350 million hectares by 2030 [44]. This initiative, later supported and broadened by the New York Declaration on Forests at the 2014 UN Climate Summit, is based on a forest landscape restoration (FLR) approach [45]. Additional initiatives include the Zero Net Land Degradation [46], which calls leaders to avoid degradation of productive lands and restore degraded lands and the Initiative 20x20 for Latin America [47], which promotes the recovery of productivity on degraded lands. In addition, in the Sustainable Development Goals [48], restoration is explicitly annotated in objective 15 (“Life on Earth”) to fight desertification and to stop and reverse land degradation. Finally, the United Nations General Assembly has declared 2021to 2030 the “Decade on Ecosystem Restoration” [49,50]. These global initiatives offer unparalleled political and economic opportunities for halting and reversing environmental degradation.

None of the global initiatives, however, include specific strategies for restoring ecosystems. Available international guidelines on implementation and monitoring are included in the Society for Ecological Restoration International Primer on Ecological Restoration [51], the International Principles and Standards for the Practice of Ecological Restoration [14,52], and the “Practitioner’s Guide” of the International Union of Forest Research Organization [53]. To face the multiple challenges of ecosystem restoration, it is important to analyze information about the strategies implemented on the ground as well as the results and monitoring scheme used to evaluate those results. Here, we systematically analyze the information obtained from a national assessment of 75 projects performed in Mexico, including the following: (i) technical aspects and the interventions executed, (ii) ecological and socioeconomic results, and (iii) monitoring of the actions implemented, including criteria and the person responsible for carrying it out. Understanding how restoration actions are conducted allows the identification of gaps in implementation and thus the design of policies and prioritization of studies needed to improve restoration strategies [54]. Moreover, information about the interventions performed, the results of restoration, and the variables monitored are fundamental for scaling and prioritizing actions to the landscape level [31].

## Materials and methods

Data were collected between 2015 and 2016. Six complementary procedures were used to identify the restoration projects: (1) a Google search using the keywords restaur*, recuper*, restor*, recover* *México* and *vegetación*; (2) direct consultations with restoration practitioners and active conservation institutions; (3) review of conference abstracts available since 2000 from the meetings of the Botanical Society of Mexico, AC, the Mexican Scientific Society for Ecology, the Society for Ecological Restoration held in Mexico and the first Mexican Ecosystem Restoration Symposium in 2014; (4) search for on-line documents from institutional and academic libraries; (5) seek information on restoration projects in specialized databases; the search was performed in the Global Restoration Network, EcoIndex, and the databases of the Commission for the Knowledge and Use of Biodiversity (CONABIO), the National Institute of Ecology and Climate Change (INECC), and the Mexican Network for Environmental Restoration (REPARA) and (6) consultation of the Mexican Conservation Board to identify individuals, academic and government institutions, and civil society organizations with a mission that included ecological restoration [55]. This allowed the identification of 293 entities involved in ecological restoration actions. Through this ample search, we identified a sample of 188 projects, which was later reduced to 150 projects after excluding projects in marine or aquatic environments and those for which only information from the diagnostic stage was available. Projects were also excluded from analysis when no technical reports were available or when we were unable to contact the project manager by email or phone. The projects were also ineligible when the survey was not completed during the dates available or when institutions did not have documents about the projects.

The information on the restoration projects was collected through a semi-structured digital survey via LimeSurvey ver. 2.65.0 (https://www.limesurvey.org) or was extracted from available technical reports. This survey was adapted to the Mexican context from the assessment protocol for restoration projects designed by Murcia and Guariguata for Colombia [41]. The survey consisted of open and closed multiple choice questions. The survey was sent to people involved in the 150 projects mentioned above. All the participants in the survey were given an information sheet about the project and asked to provide informed consent in writing. Research was approved by the Universidad Autónoma del Estado de Morelos and the Instituto de Ecología, A.C. and in compliance with its code of research integrity. Additionally, people and institutions were contacted by phone to verify details. The survey was open for three months, after which we received information from 58 projects. For an additional 17 restoration initiatives, information was obtained from published technical reports. It was not always possible to obtain information for all the fields of the survey; thus, sample sizes varied among variables. In results, we report the number of cases containing full information at each section. The answers were accepted as *bona fide* as no field visits were made to corroborate the accuracy or veracity of the reported data.

The complete list of projects and the entire survey composed of 137 questions can be consulted in the following publication: La restauración de ecosistemas terrestres en México: Estado actual, necesidades y oportunidades [56]. Here, we analyze information from questions available in S1 Appendix, which addressed the following: (i) the technical aspects of the interventions executed, (ii) ecological and socioeconomic results and (iii) monitoring actions, e.g., type of monitoring and responsible parties. The section of technical aspects compiles information on the type of intervention, source of the techniques implemented to recover flora and fauna, and the origin of biological material. The section of ecological results explores progress towards targets and goals regarding initial conditions and the reference ecosystem. The section of socio-economic results examines individuals’ perceptions of collaboration among organizations, individuals, and institutions. We also included in this section whether because of restoration there was an application or creation of economic incentives. Finally, questions in the section of monitoring actions gather information about planning, variables used, monitoring type, and the identity of the executors, as well as whether adaptive management was included. The responses from open questions were reduced to categorical variables measured in the short-, middle-, and long-term. The information of projects was organized in Excel® spreadsheets and further processed using the "plyr", "dplyr", "tibble" and "tidyr" libraries of the free access R environment [57].

## Results

### Technical aspects of execution

#### Interventions carried out

Fifty-nine of the projects (79%) were developed in terrestrial ecosystems, while only 16 (21%) were in wetland ecosystems. Of these, 12 (75%) gave information on the interventions carried out (Table 1). For 44% (N = 33) of the projects, civil infrastructure works were required before implementing restoration actions. Minimal intervention was the most frequent restoration action in 97% of terrestrial ecosystems. Disturbance exclusion (78%) to foster natural succession (77%) was the most frequent restoration strategy at this level of intervention. Maximal interventions were in second place, and plantings of species with specific ecological attributes (61%) and mixed tree plantings (47%) were the most frequent restoration strategies at this level of intervention. Intermediate interventions were mentioned in only 31% of projects, including eradication of alien and/or invasive species (61%) and nucleation, which were the preferred implemented actions in this category (44%).

**Table 1:** The frequency (percentage) of actions included in the restoration projects classified as minimal, intermediate, or +maximal intervention level.

Level of intervention/Restoration actions	Frequencies (%)
**Terrestrial ecosystems**	
***Minimal***	**57 (97)**
Disturbance exclusion (e.g., livestock exclusion) to foster natural regeneration	44 (77)
Re-establishment of fire regime (i.e., control of fires or controlled burnings)	13 (28)
***Intermediate***	**18 (31)**
Eradication of alien and/or invasive species to favor natural regeneration	11 (61)
Nucleation (i.e., supports, wildlife refuges, transfer of soil)	8 (44)
Herbicides application to remove competitive species	3 (17)
Pollutants control	1 (6)
Others (e.g., seeding native grasses)	2 (11)
***Maximal***	**51 (86)**
Plantings of species with specific ecological attributes (i.e., nitrogen fixers, habitat providers)	31 (61)
Mixed tree plantings	24 (47)
Mixed plantings of trees, bushes and/or herbaceous plants	18 (35)
Establishment of structures for fauna colonization	14 (27)
Re-introduction or re-location of fauna	7 (14)
Monospecific plantings with species different from the potential ecosystem	6 (12)
Fertilization inputs for increasing plant performance	5 (10)
Bioremediation to reduce soil or water toxicity	1 (2)
Others (i.e., monospecific plantings with local species, recovery of substrate or removal of exotic fauna)	4 (8)
**Wetlands ecosystems**	
***Minimal***	**10 (83)**
Promoting natural regeneration	9 (90)
Re-establishment of the hydrological regime (e.g., dyke removal, channel opening)	8 (80)
Sediment removal	2 (20)
***Intermediate***	**6 (50)**
Transfer of sediments and/or seed banks	5 (83)
Other (i.e., removing invasive alien species or stopping fishing)	1 (17)
***Maximal***	**8 (67)**
Sowing or transplanting emerging plant species (i.e., reeds or rushes)	8 (100)

Within one project, multiple actions can take place for each intervention level; multiple intervention levels can be implemented in one restoration project. Boldface figures indicate the number of projects that implement minimum, intermediate or maximum interventions. The total number of projects analyzed was 75.

In 83% of wetland ecosystems, minimal interventions were the most frequently implemented intervention (Table 1). Ninety percent of projects included promoting natural succession and in 80% the re-establishment of a hydrological regime was required. Intermediate interventions were the second most common in wetland ecosystems; 42% of projects required the transfer of sediments and/or seed banks. Finally, maximal intervention was established in 67% of the projects and included the sowing or transplanting of emerging plant species.

#### Source of implemented techniques

The techniques used in restoration processes were most frequently developed for the project and executed by their creators (80%; N = 69); some were taken from previously developed projects (36%). Collective learning (through a knowledge dialogue with local people) was not a particularly frequent source of implemented techniques (38%; traditional management). To a lesser degree, techniques were adapted from national (23%) or international literature (13%), and rarely were the used, protocols given by the convener or contracting institution (12%).

#### Criteria used for selection of biological material and its source

**Fig 1:**
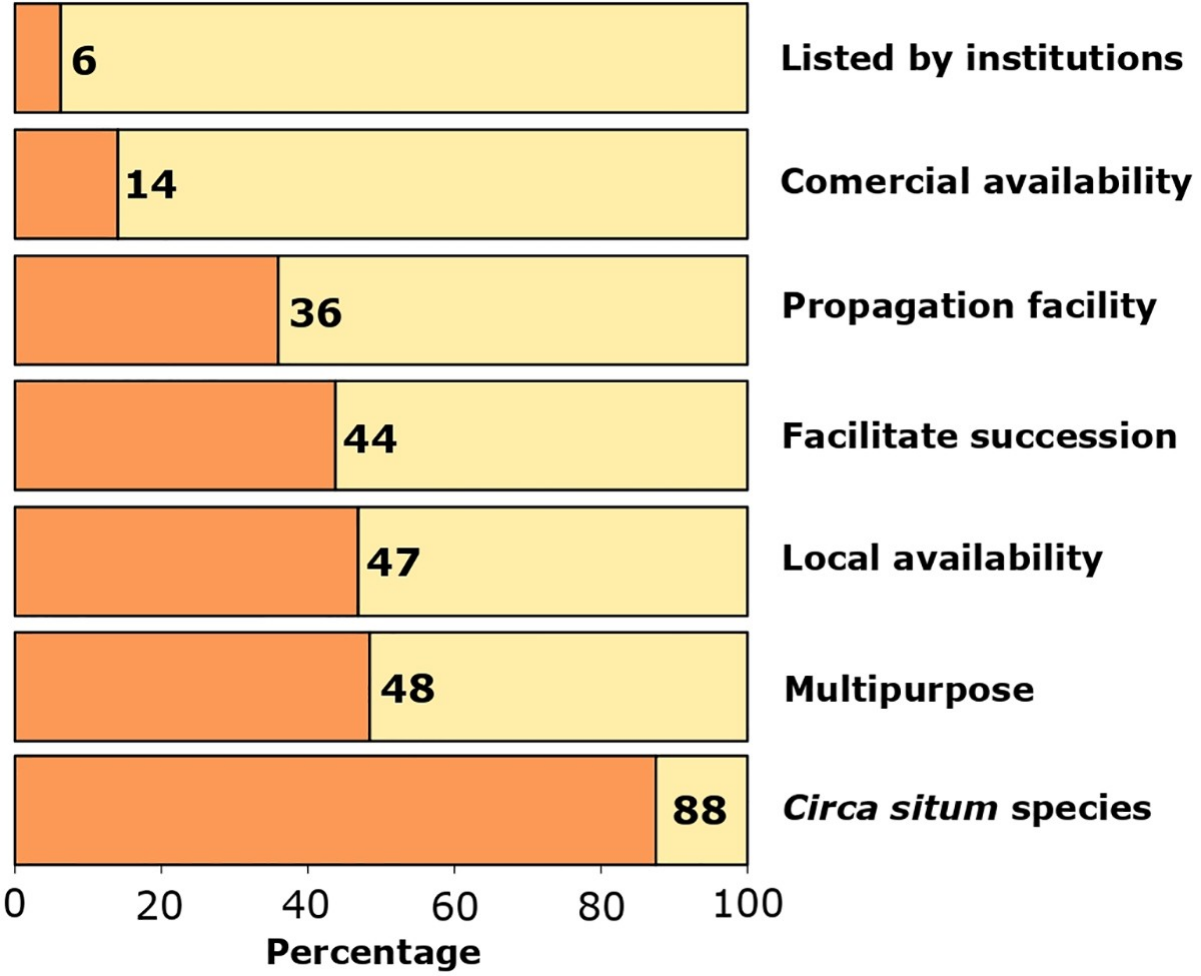
Criteria used for biological material selection in the restoration projects. (i) *circa situm*: Species within to the restoration zone (natives or naturalized), (ii) multipurpose species, (iii) local availability of seeds and seedlings (extracted from reference neighborhood or nearby sites), (iv) species that facilitate succession (nursery plants or catalyzing species, for example, species producing fruits that feed local fauna, nitrogen fixers, soil fixers, etc.), (vi) ease of propagation or reproduction, (vii), commercial availability of seeds or germplasm, and (viii) listed species by convener institutions. The total number of projects analyzed was 64.

Biological material selection was most often multicriterial (Fig 1). In 88% of projects, the biological material was selected under a *circa situm* criterion, i.e., that species were native or naturalized to the restoration zone. Forty-eight of the projects selected multipurpose species, and 47% considered the local availability of seeds or seedlings. In 44% of the projects, species that facilitate ecological succession were selected (Fig 1). Ease of propagation or reproduction (36%), commercial availability of seeds or germplasm (14%) or species’ inclusion on lists generated from government or other institutions (6%) were not frequent criteria.

With respect to the source for acquiring the biological material used, in 48% (N = 64) of the projects, implementers propagated the material themselves, while in 40% of the projects, the material was purchased from local nurseries. Twelve percent of the projects mentioned other sources, such as the extraction of seedlings from nearby sites or rescue from sites that were slated for exploitation.

### Ecological, social, and economic aspects of restoration projects

**Fig 2:**
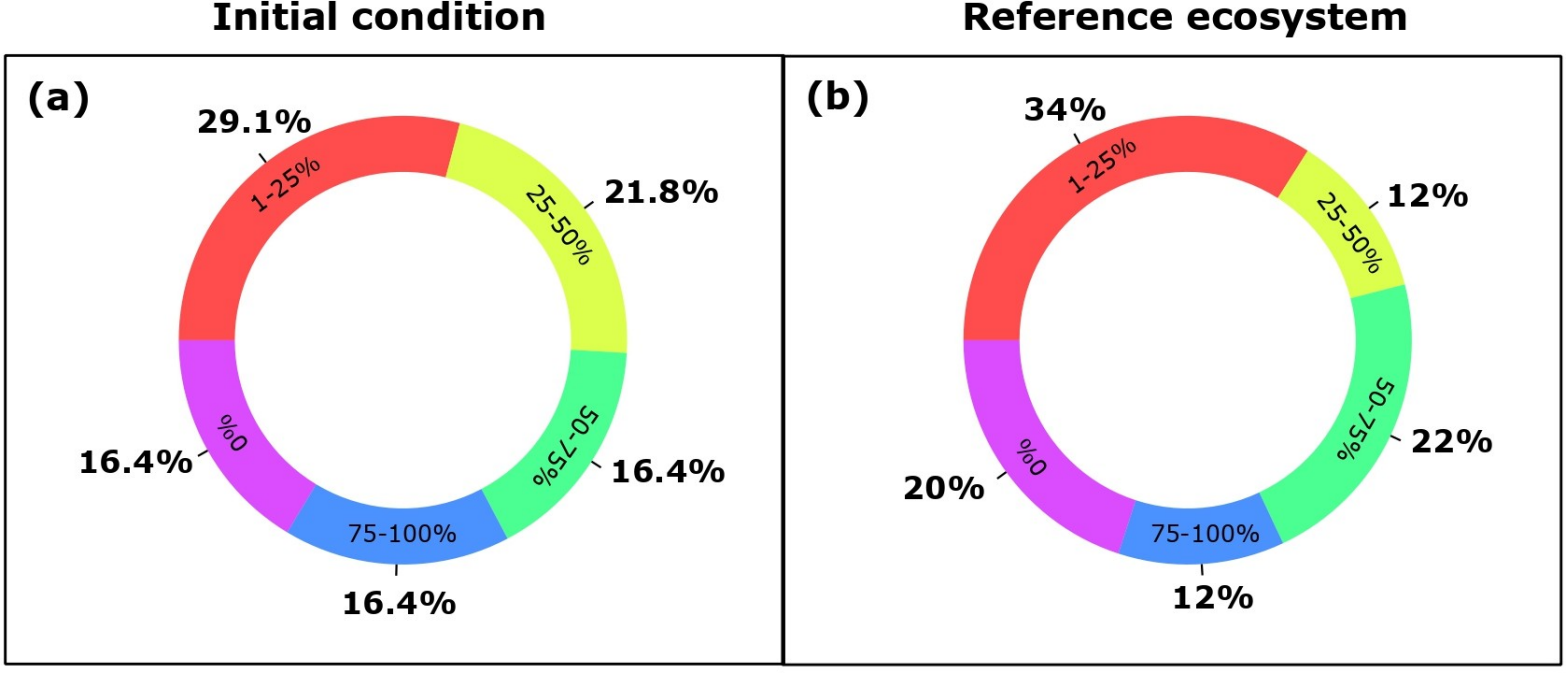
Recovery of biodiversity. Percentage of projects in each of four class types (inside the donuts) indicating degree of biodiversity recovery compared with (a) the initial condition of the ecosystem and (b) a reference ecosystem. The total number of projects analyzed was 55 in (a) and 50 in (b).

Only a few projects reached a high level of biodiversity recovery (Fig 2). For example, in 29% of the projects, recovery of biodiversity was between 1 and 25% relative to the initial conditions (Fig 2A), and only 16% of projects claimed 75 to 100% recovery. The situation was similar considering the biodiversity recovery relative to a reference ecosystem (Fig 2B).

The recovery of provisioning ecosystem services was less than 50% in 69% of projects when using the initial ecosystem conditions as the reference point (Table 2). When recovery of services was compared with the reference ecosystem, the percentage of projects increased (71%). Most of the projects recovered less than 50% of regulating services regardless of whether the comparison was with the initial ecosystem conditions or with the reference ecosystem.

**Table 2:** Ecosystem services recovery.

Service	Recovery degree					
	0%	1–25%	25–50%	50–75%	75–100%	N
**Initial conditions (N = 52)**						
Provision	6 (17)	12 (33)	7 (19)	4 (11)	7 (19)	36
Regulation	8 (20)	8 (20)	9 (23)	5 (13)	10 (25)	40
**Reference ecosystem (N = 53)**						
Provision	8 (21)	14 (37)	5 (13)	6 (16)	5 (13)	38
Regulation	10 (24)	10 (24)	7 (17)	7 (17)	7 (17)	41

Number of projects (percentage) in each of the five classes of recovery degree of ecosystem services regarding the initial conditions of the ecosystem or the reference ecosystem. The number of projects and the percentage in parenthesis is shown in relation to the total number of projects that considered ecosystems services (N). Provisioning services: Any type of benefit to people that can be extracted from nature including food, drinking water, firewood, fiber, chemical or biological products or genetic resources. Regulating services are the benefit provided by ecosystem processes that moderate natural phenomena and include climate regulation, disease regulation, water regulation, regulation associated with biodiversity.

**Fig 3:**
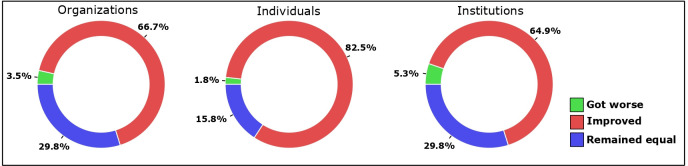
Social results of restoration. The chart shows the percentage of projects where an improvement, a setback or a similar collaboration was perceived in ecological restoration actions among non-governmental organizations, individuals and scientific or educational institutions. The total number of projects analyzed was 57.

For 82% of the projects, it was perceived that relationships among individuals improved, as did collaboration among non-governmental organizations (67%) and scientific or educational institutions (65%; Fig 3).

In most of the projects, no monetary incentives were applied or created (52%; Table 3). Moreover, only 8% requested payment for ecosystem services, and only 4% of projects applied voluntary market mechanisms such as a carbon credit. The employment of local community members for the implementation of projects was an incentive that was not included among multiple choice options but was mentioned in open responses by surveyed individuals (25%).

**Table 3:** Socio-economic results of restoration.

Variables	Frequencies (%)
No monetary incentives were applied or created	37 (52)
Employment of local communities	18 (25)
Payment for ecosystem services	6 (8)
Applied voluntary market mechanisms	3 (4)

The frequency (percentage) of socio-economic variables included in the restoration projects. The total number of projects analyzed was 71.

### Monitoring practices in restoration projects

In 43 projects (57%), a monitoring plan was established *a priori* (N = 75). Among these, 20 (47%), indicated that there was funding set aside for monitoring actions. Only 35 (81%) projects provided information on the frequency of evaluations. Annual evaluations were the most frequent (51%; 18 cases), followed by evaluation every six months (23%; 23 cases), every three months (20%; 7 cases), monthly (17%; 6 cases) and every two months (9%; 3 cases).

Generally, variables from more than one category were used for monitoring (Table 4). Most of the projects (88%) monitored several short-term changes and one long-term variable (88%). These include plant survival and growth, changes in vegetation structure (74%), environmental and physicochemical parameters of water, such as site quality or ecosystem water quantity (28%), indicators of erosion control (23%), and vegetation cover (23%). Medium-term changes, like colonization of fauna (21%), carbon accumulation, ecosystem productivity or soil nutrients increases (14%), and secondary succession (12%) were assessed in 40% of the projects. The only long-term variable—anthropic perturbation and human settlements—was measured in 88% of projects. Social changes, i.e., community perception of social or environmental benefits of the restoration project, were monitored in only 2% of projects (Table 4).

**Table 4:** Monitored variables.

Monitored variable	Frequencies (%)
**Short term**	**38 (88)**
Plant survival and growth, and vegetation structure	32 (74)
Site quality/water quality	12 (28)
Erosion control/organic litter accumulation	10 (23)
Vegetation cover	10 (23)
Vegetation composition	5 (12)
Control of invasive species	1 (2)
**Middle term**	**17 (40)**
Wildlife species and monitoring	9 (21)
Carbon/nutrients/productivity	6 (14)
Secondary succession	5 (12)
Seed dispersal	4 (9)
Reproductive status of plants	2 (5)
Habitat for fauna	1 (2)
**Long term**	**38 (88)**
Control of disturbances/presence of settlements	38 (88)
**Social**	**1 (2)**
Community perception (i.e., social, or environmental benefits due to restoration projects.	1 (2)

The table shows the frequency (percentage) of the variables included in the monitoring phase of restoration projects. The total number of projects analyzed was 75.

**Fig 4:**
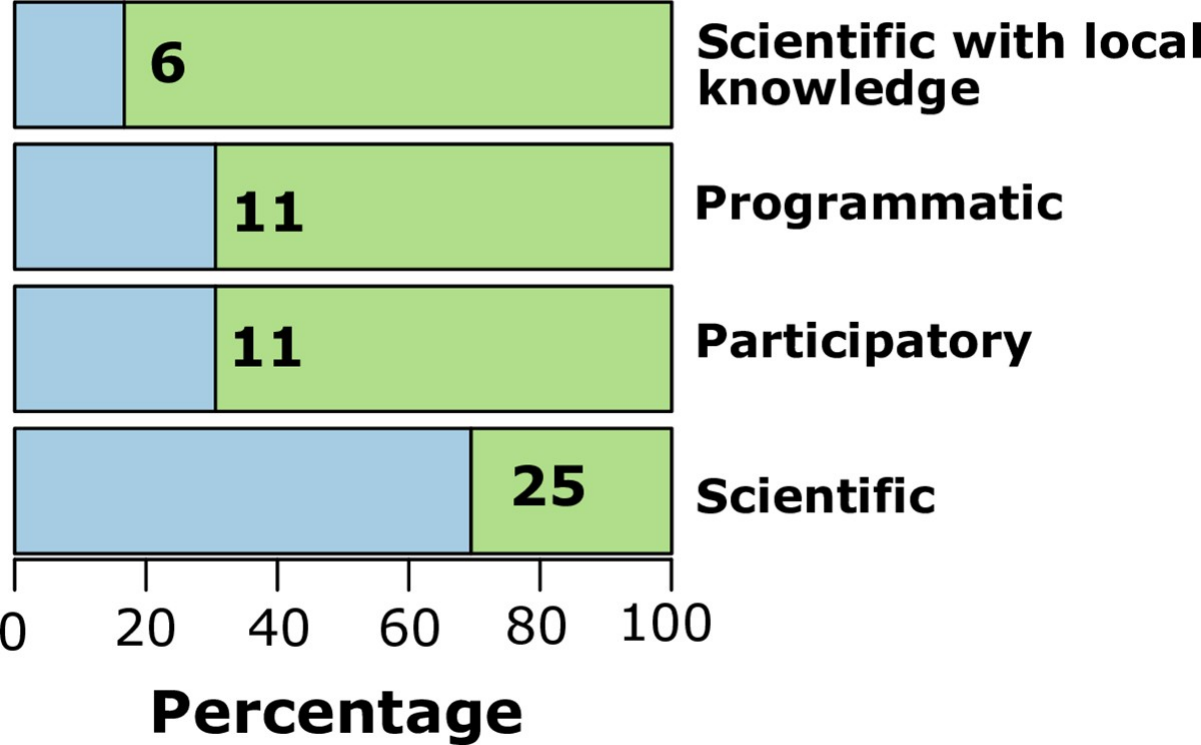
Monitoring types used to measure the success of ecological restoration actions. The total number of projects analyzed was 36.

For 69% of the projects, the scientific method was the most frequently used to measure success, progress, or effectiveness of actions. Programmatic monitoring (i.e., monitoring activities laid out *a priori* at specific times during the development of the project) and participatory (collaborative) monitoring were used in equal percentages (31% each). Scientific monitoring with local knowledge was the least used method to measure success (17%) (Fig 4).

In most of the projects, monitoring was led by academics (54%; Table 5). Community members participated in monitoring in 32% of projects. Government institutions (32%) and academics (24%) handled the legal part (established by law) of the monitoring phase. Financial resources for monitoring were mainly provided by the government (70%) and academic sector (38%). The private sector was only rarely responsible for the technical or legal aspects of project monitoring (6 and 5%, respectively), and community members only rarely participated as funders (6%). No international institutions were the main parties responsible for the technical, legal, or financial aspects of restoration projects’ monitoring.

**Table 5:** Role played by stakeholders of society in the monitoring of restoration projects.

Variables	Frequencies (%)
**Technical managers**	
Academy	22 (54)
Community members	13 (32)
Non-Governmental Organization	12 (30)
Government	11 (28)
Landowners	11 (28)
Private (companies or entrepreneurs)	2 (6)
**Legal responsible**	
Government	13 (32)
Academy	10 (26)
Landowners	9 (22)
Community members	7 (18)
Non-Governmental Organization	6 (16)
Private (companies or entrepreneurs)	3 (8)
**Financial managers**	
Government	28 (70)
Academy	15 (38)
Landowners	8 (20)
Non-Governmental Organization	7 (18)
Private (companies or entrepreneurs)	4 (10)
Community members	2 (6)

Frequency (percentage) of different stakeholders involved in the monitoring of restoration projects. The total number of projects analyzed was 41.

Information related to adaptive management was gathered from 27 projects, of which 16% reported that they had not yet analyzed results, 19% reported that they already had results showing the need to adjust but had not yet implemented the required changes, and 7% reported a diagnosis and that they were making adjustments.

## Discussion

### Ecological restoration on the ground

Minimal intervention such as disturbance exclusion to foster natural regeneration or re-establishing natural fire regimes were the preferred implementation approaches. All of these actions involve removing disturbance from an ecosystem [1,58] to allow natural succession to begin [59]. Favoring natural regeneration can potentially be applied to very large areas [4]. For example, unassisted regeneration has been the main strategy implemented in large scale-restoration projects (>100,000 ha) in Latin America [60]. Restorations methods based on natural regeneration are considerably less expensive than tree plantings, making it more cost-effective on a large scale [61]. For instance, the restoration of more than 3000 ha in the state of Rio de Janeiro, Brazil, would have cost US $15.1 million using tree plantings [62]. Although natural regeneration is frequently adopted because of the usually limited resources in the restoration projects [41,56], their implementation should consider the potential for natural regeneration, and the landscape and social context [63]. Overall, the potential for natural regeneration increases in agricultural landscapes when the size, duration, and severity of disturbance [64,65], or degree of transformation is low [66]. Natural regeneration potential is also low in climatic hardness conditions [temperature and vapor pressure deficit; 64]. At the landscape scale, the most critical factors for a high natural regeneration potential are the proximity to existing forested areas and the dispersal capacity of species [67]. Lastly, human development is also a predictor of the potential for natural regeneration since the lowest recovery of biodiversity by natural regeneration is likely to occur in countries with an intermediate rating Human Development Index [HDI; 66]; the HDI is based on life expectancy, education, and income (http://hdr.undp.org/en/content/human-development-index-hdi). Opting for minimal intervention actions is viable when the natural regeneration potential is high. Thus, it is fundamental to evaluate natural regeneration potential *a priori* to deciding the level of intervention to apply.

Restoration plantings are frequently implemented in restoration projects. Although this method requires a large amount of financial resources [7] and prior knowledge of plant performance to be successful, its implementation removes several biotic (e.g., competition, dispersal limitation) and abiotic (e.g., environmental conditions) barriers to natural succession [10]. Restoration plantings are generally established in small-scale projects and/or in heavily degraded landscapes [68]. In Mexico, 67% of the projects were small in scale (< 1,000 ha, mean = 94.6 ha), and the most frequently identified threats were extensive cattle ranching and fragmentation. In addition, the majority of the projects were financed by the government [56,63]. These results from a previous analysis of the characterization of projects in Mexico suggest that most of the restoration initiatives were in highly degraded and fragmented areas, which would explain why maximal intervention was also heavily represented there. Although restoration plantings are usually expensive [6], their implementation in Mexico was frequently financed and promoted by government institutions such as the National Forestry Commission [69]. A similar situation has been found in Colombia, in which most of the interventions are on an even smaller scale (< 100 ha, mean = 29 ha). The main threats are cattle ranching and agriculture in fragmented ecosystems; furthermore, the most frequently used restoration technique is the plantings of trees and shrubs, and a great proportion of projects are government financed [41].

An alternative to these restoration strategies is assisted natural regeneration (ANR). ANR reduces or eliminates the costs associated with propagating, raising, and planting, making it a simpler, less expensive, and usually effective technique for recovering forest productivity [8]. Eradication of alien and/or invasive species, nucleation, herbicides application and seeding were intermediate interventions applied to a lesser extent. ANR strategies are often used to remove some of the biotic barriers to natural succession and therefore accelerate recovery [6]. For example, alien or invasive species usually compete intensely with local species for resources, slowing down or arresting natural succession [70]. To eliminate this barrier, competing plants can be removed manually, with tools (e.g., machetes), or by applying herbicides [71,72]. These ANR strategies are generally used in an adaptive management context in conjunction with other levels of intervention or are targeted to solve a particular problem. For example, the proliferation of climbing plants in early successional habitats is a frequent phenomenon that limits the development of plants [72,73]. In both tropical and temperate forest, ruderal climbing plants or woody lianas are favored by disturbance, and affect tree growth and forest biomass and hence arrest forest succession [74,75]. One way to facilitate forest regrowth is by removing those plants [76–78]. ANR represents an alternative way for recovering degraded ecosystems at intermediate levels of degradations at a reasonable cost.

As one might expect, minimal interventions were the preferred restoration strategy in wetlands. Changes in hydrology have been identified as one of the main causes of mangrove degradation [79]. Therefore, to foster mangroves natural regeneration hydrological rehabilitation is implemented [80]. Mangroves can regenerate naturally if the tidal hydrology has not been disrupted and if there are sufficient mangrove propagules nearby [81]. Performing minimal cost-effective interventions such as hydrological rehabilitation, where degraded and destroyed channels are reconnected, can allow fast natural regeneration of mangroves [82,83]. For hydrological recovery, actions must target the same slope and elevation as the reference mangrove forest [83]. Before deciding wetland ecosystem interventions, however, an environmental characterization and diagnosis of stressors causing its degradation are needed [84]. The cost- effective ecological restoration of wetlands requires the recovery of hydrology and the identification of ecosystem factors stressors.

### The need for transdisciplinary (not only multidisciplinary) approaches

Traditional management practices remain an undervalued source of knowledge for restoration. Our results indicate that top-down approaches are still common in restoration action and policies (decision-making). Some authors have indicated that Traditional Ecological Knowledge (TEK) is poorly applied in ecological restoration programs [17–19], including in several Latin American countries [60,85]. The participation of Indigenous Peoples and Local Communities (IPLCs) has been limited to the execution of actions (e.g., field labor) instead of co-designing restoration projects together with IPLCs that build on their TEK and experiences [86]. It is also evident that TEK is still less considered in planning and monitoring [15] and that IPLCs are still absent in environmental policy forums at the country, regional and worldwide levels [19]. In future restoration projects, incorporating TEK from IPLCs in the stages of planning, execution and monitoring will be fundamental for the recovery of ecosystems. Transdisciplinary research practices will likely be important in this context because they highlight the early involvement of non-academic actors in research projects, including ecological restoration projects. For instance, during the planning stage, IPLCs can supply reference ecosystem information. Similarly, IPLCs can inform about key species [87] and cultural places (e.g., food, medicine and foraging species) that make up the basis of a culture [88]. They also might advise about particular places that are of critical importance for the flow of ecosystem services and way of life [89]. During the execution of projects, IPLCs may contribute to (re)orienting the processes of ecosystem restoration. For instance, IPLCs have plenty of experience in the traditional management systems that can help or speed up succession [90–92]. Other TEK-based land management practices, like rotational swidden cultivation systems, agroforestry, fallow and culture improvement systems, hedgerow implementation, and living fences, have also been shown to be effective in carbon sequestration and fighting environmental degradation and desertification [93,94]. Finally, although the contribution of TEK to the progress of restoration is still insufficiently acknowledged, this source of knowledge can also be useful in designing and implementing restoration monitoring programs [16]. Local communities often have a clear understanding of the factors that threaten their territory and cultures and thus can tell us a lot about which variables to measure [92]. The application of TEK in ecological restoration can help inform site and species selection for restoration and provide historical information on ecosystem’s state (ecosystem reference) and land management.

### Adequate planning for enhancing restoration success

Only a small fraction of projects that report ecological results (biodiversity) claimed to have achieved most of their planned goals. This result contrasts with the fact that more than 80% of the projects said to have defined their reference ecosystem [63]. A restoration project will have better transparency, management capacity and chance of success if the ecological goals and objectives are clearly defined, can be easily measured, adequately evaluated, and planned based on a reference ecosystem [52]. The lack of adequate planning for the accomplishment of the goals seems to be a generalized practice in Latin America [85,95], and reflects a lack of forethought about the factors that can influence the achievement of the goals, such as the depletion of funds [11,39] or socioeconomic reality. Similarly, it makes evident the deficiency in planning based on a clear theoretical framework to achieve short-, medium-, and long-term goals.

Evaluating the results of ecological restoration projects is not an easy task to achieve. This is because in some restoration projects the desired results can take several decades to become evident [96]. In addition, post-disturbance ecosystems may have moved into alternate states [97]. An, *a priori* diagnosis of the natural regeneration potential and identification of key factors that limit or determine the recovery of the ecosystem subject to intervention, however, is a fundamental step for obtaining satisfactory results [14,52], such as the recovery of biodiversity and associated ecosystem services [98,99]. These evaluations could be performed using low-cost and time-efficient indices or methodologies reported in the literature [64,100,101]. These evaluations, however, were apparently absent from the restoration projects. Scientists as well as land managers and the public might be able to assess initial ecosystems state using ecological indices in order to identify adequate restoration actions; see the Ecological Disturbance Index for tropical areas [7]. This absence of initial evaluation, perhaps because of lack of technical capability or insufficient budgeting for developing baseline assessment as we found preciously [63], could explain the low levels of recovery of biodiversity and ecosystem services reported in the restoration projects. Having a clear understanding of the importance of adequate planning and initial evaluation would maximize the available funds and restoration results.

The success of restoration projects is not only a matter of ecological results. It is important to know whether projects lead to positive interactions among individuals. Despite the low levels of recovery of biodiversity and ecosystems services reported in most of the projects, positive interactions among local people were identified. This may be because individuals’ confidence in restoration activities is reinforced by the knowledge that others are also participating [102]. This positive interaction among community members increases cooperative spirit and reduces project costs; rather than hiring people outside the restoration areas, community participation along with adequate technical training could lead to improved restoration results [103]. Overall, social participation was adequate; however, this was limited to the field actions [86]. Moreover, we previously found that field work, (i.e., implementation, monitoring and maintenance) is more frequently considered in the budget (74%) than desk work (50%; [86]). Restoration results would benefit from the inclusion of community members beginning at the decision-making and planning stages, and not just as the work force during the execution stage [86]. Social involvement in steps other than field work is crucial for ecosystem recovery on a large scale.

In general, environmental services payments were not included in the restoration activities. This would be due to the uncertainty of the continuity of the project in the long term as a result of financial problems [104]. For example, in the Mexican and Colombian national country-wide assessment of ecosystems restoration projects, 39 and 29 projects, respectively identified financial limitations [41,56] Moreover, environmental services payment has a limited efficacy in promoting restoration [105]. Environmental services’ payment schemes are most efficient when efforts are directed to only one service, like carbon capture [106]. This is usually a problem because most projects are multifunctional, and the actions are oriented toward biodiversity recovery.

### An urgent need for monitoring the progress of ecological restoration

Although some monitoring protocols were established *a priori*, they were considered an extra cost of projects rather than a necessary investment. In Mexico, a previously analysis on the planning phase of restoration found that only 22 projects of 75, considered the monitoring in the budget [63]. Insufficient budgeting for the monitoring phase may be due to the limited amount of money allocated to assess progress of restoration by governments that focus mostly or exclusively on implementation. Furthermore, the monitoring practices are usually not allowed in ecological restoration proposals [63]. Monitoring is an essential component of recovery projects because of to their long-term nature [107]. Monitoring enables the evaluation of restoration progress and informs subsequent management decisions and the adoption of corrective measures [108,109]. Information about monitoring is essential for land managers who need evidence-based evaluations to assess the achievement of restoration actions [110]. Monitoring helps to verify whether specific endpoints have been reached, determine which restoration strategies are best suited for the recovery of a process, biodiversity, or an ecosystem service [111]. In addition, the evaluation of restoration progress serves as a means for social learning [112]. For example, besides of quantifying the number of hectares planted, monitoring can inform restoration practitioner about increases in forest cover [e.g., 32], or whether a recovered area is supplying the expected environmental and social benefits [113,114]. In the long term, monitoring helps to determine which species combinations can resist invasion and whether plant communities with complementary or redundant traits confer greater invasion resistance [115,116]. Monitoring protocols need to be carefully planned and be part of a project from its conception if they are to secure the long-term sustainability of restored areas.

According to our data, monitoring generally takes a top-down approach and is focused mostly on short-term ecological indicators. Local community members only rarely participated in monitoring or were the main responsible parties. National inventories of restoration in Colombia [41] and Peru [117], as well as the Latin American regional evaluation of integrated landscape initiatives [118], showed similar results in terms of community participation. Including IPLCs in a participatory monitoring scheme [39] can be useful for designing and implementing restoration monitoring programs [16]. A recent study [39] showed that local people accurately collect data on forest change, drivers of change, threats to reforestation, and biophysical and socioeconomic impact. This valuable information might be difficult to obtain by other means. Several initiatives that included IPLCs in the monitoring of carbon capture are gaining importance because of the efforts of REDD + (Reducing Emissions from Deforestation and forest Degradation, +, as well as conservation and sustainable development) [e.g., 119,120]. In Mexico, through the Program for the Conservation of Species at Risk [PROCER; 121], IPLCs are involved in monitoring restored sites within natural protected areas. Although the incorporation of IPLCs in some monitoring processes is becoming more common [15], in most of the 75 projects that we evaluated, short-term ecological indicators were used in monitoring. Similar results are documented in Colombia, where 96% of the projects only monitored only short-term changes, such as the early survival and growth of planted seedlings, changes in plant ground cover, and erosion control [85]. To date, 16 Latin American countries have committed to ecological restoration [47], but only four countries had developed national or subnational strategies by 2016 to implement their national restoration targets [122]. Fulfilling those commitments and scaling up restoration actions will require the inclusion of IPLCs in restoration programs at all stages—from planning through implementation, through monitoring.

## Supporting information

S1 Appendix Survey used to collect the data. (DOCX)
