# Supplementary material for: Challenges during the execution, results, and monitoring phases of ecological restoration: Learning from a country-wide assessment
Source: PLoS One. 2021 Apr 6;16(4):e0249573. doi: 10.1371/journal.pone.0249573 (PMC8023452; doi:10.1371/journal.pone.0249573)
Supplement: S1 Appendix — (DOCX) [file pone.0249573.s001.docx]

**S1 Appendix**. Questions analyzed in this paper:

**1.1. Interventions carried out and source of implemented techniques.**

1.- What kind of interventions were or have been necessary for the control of disturbance factors?

2.- What kind of interventions were or have been necessary for the restoration of flora?

3.- What kind of interventions were or have been necessary for the restoration of flora?

4.- What kind of interventions were or have been necessary for the restoration of native fauna?

5.- Who developed the implemented techniques?

**1.2 Source of biological material and criteria used for its selection.**

6.- What criteria were used to select species?

7.- Do you know where the organisms used in the project come from?

**2.1. Recovery of biodiversity**

8.- To date, how much has biodiversity (e.g., abundance, species richness, biomass) recovered from the initial conditions of the ecosystem?

9.- To date, how much has biodiversity (e.g., abundance, species richness, biomass) recovered from the conditions of the reference ecosystem?

**2.2. Progress on ecosystem services recovery**

10.- If ecosystem services were part of the project's goals, to date:

10.1 How much have PROVISION services recovered from the initial conditions of the ecosystem?

10.2 How much have REGULATION services recovered from the initial conditions of the ecosystem?

11.- If ecosystem services were part of the project's goals, to date:

11.1 How much have PROVISION services been recovered with respect to

the conditions of the reference ecosystem?

11.2 How much have REGULATION services recovered from

to the conditions of the reference ecosystem?

**2.3. Socio-economic results of restoration**

Collaboration between:

12.- ORGANIZATIONS involved in different phases of the project have improved, worsened or remained the same.

13.- INDIVIDUALS who participated in different phases of the project has improved, worsened or remained the same.

14.- INSTITUTIONS that participated in different phases of the project has improved, worsened or remained the same.

15.- Derived from the restoration project, were some of the following socio-economic incentives implemented or created?

Employment of local communities

Payment for ecosystem services

Applied voluntary market mechanisms.

No monetary incentives were applied or created.

**3. Monitoring practices in restoration projects**

16.- It was developed *a priori* plan to follow (monitoring) and assess the effects of intervention?

17.- Indicate the temporality of assessments.

18.- ¿Funding for monitoring actions was reserved since project conception?

19.- What variables were used to measure success, progress, or effectiveness? List all the ones you are monitoring.

20.- What kind of monitoring is used to measure the success, progress, or effectiveness of actions?

21.- Who are the technical managers to follow up on restoration processes?

22.- Who is responsible for following up on actions?

23.- Who are the funders of follow-up actions?

24.- Have you performed adaptive management actions based on monitoring?
